# Using intranasal dexmedetomidine with buccal midazolam for magnetic resonance imaging sedation in children: A single-arm prospective interventional study

**DOI:** 10.3389/fped.2022.889369

**Published:** 2022-08-04

**Authors:** Bi Lian Li, Hao Luo, Jun Xiang Huang, Huan Huan Zhang, Joanna R. Paquin, Vivian M. Yuen, Xing Rong Song

**Affiliations:** ^1^Department of Anesthesiology, Guangzhou Women and Children’s Medical Center, Guangzhou Medical University, Guangzhou, China; ^2^Department of Anesthesiology, Cincinnati Children’s Hospital Medical Center, Cincinnati, OH, United States; ^3^Department of Anesthesiology and Perioperative Medicine, Hong Kong Children’s Hospital, Hong Kong, Hong Kong SAR, China

**Keywords:** dexmedetomidine, midazolam, children, sedation, magnetic resonance imaging

## Abstract

**Objective:**

Although numerous intravenous sedative regimens have been documented, the ideal non-parenteral sedation regimen for magnetic resonance imaging (MRI) has not been determined. This prospective, interventional study aimed to investigate the efficacy and safety of buccal midazolam in combination with intranasal dexmedetomidine in children undergoing MRI.

**Methods:**

Children between 1 month and 10 years old requiring sedation for MRI examination were recruited to receive buccal midazolam 0.2 mg⋅kg^–1^ with intranasal dexmedetomidine 3 μg⋅kg^–1^. The primary outcome was successful sedation following the administration of the initial sedation regimens and the completion of the MRI examination.

**Results:**

Sedation with dexmedetomidine–midazolam was administered to 530 children. The successful sedation rate was 95.3% (95% confidence interval: 93.5–97.1%) with the initial sedation regimens and 97.7% (95% confidence interval: 96.5–99%) with a rescue dose of 2 μg⋅kg^–1^ intranasal dexmedetomidine. The median sedation onset time was 10 min, and a significant rising trend was observed in the onset time concerning age (*R* = 0.2491, *P* < 0.001). The wake-up and discharge times significantly correlated with the duration of the procedure (*R* = 0.323, *P* < 0.001 vs. *R* = 0.325, *P* < 0.001). No oxygen deficiency nor medication intervention due to cardiovascular instability was observed in any of the patients. History of a prior failed sedation was considered a statistically significant risk factor for failed sedation in the multivariate logistic regression model [odds ratio = 4.71 (95% confidence interval: 1.24–17.9), *P* = 0.023].

**Conclusion:**

In MRI examinations, the addition of buccal midazolam to intranasal dexmedetomidine is associated with a high success rate and a good safety profile. This non-parenteral sedation regimen can be a feasible and convenient option for short-duration MRI in children between 1 month and 10 years.

## Introduction

Maintaining an adequate depth of sedation is an important part of magnetic resonance imaging (MRI) in uncooperative children due to high noise levels, long periods of acquisition time, and relatively confined spaces. Additionally, pediatricians and anesthesiologists have been facing the challenge of providing safe and effective sedation or anesthesia services for a large-volume pediatric population with limited personnel and high patient workflow (1).

Historically, propofol has been the primary sedative for pediatric MRI, owing to its acceptable sedation level, quick onset, and rapid recovery (2). However, specific concerns have been raised regarding dose-dependent respiratory depression and hemodynamic instability (3). MRI sedation-related adverse events are reported to be 0.8%; in comparison, the incidence of serious adverse events was 4.3% in patients undergoing propofol administration for imaging studies, with 18.4% of patients requiring airway-related interventions (4, 5).

Intranasal dexmedetomidine has become a widely accepted and utilized sedative for pediatric non-invasive procedural sedation in part to its neuroprotective property. It is convenient, effective, and does not cause respiratory depression which maximizes the benefits for the pediatric population (6, 7). A meta-analysis revealed that intranasal dexmedetomidine was superior to traditional oral chloral hydrate and could provide better safety for imaging sedation (8). On the other hand, dexmedetomidine has the unique capability to create a neurophysiological state similar to non-rapid-eye-movement sleep. Even at high doses, patients sedated with dexmedetomidine are still easily roused by motion or acoustic noise (9). Intranasal dexmedetomidine can be effective in 30 and 70% of children for MRI sedation at doses of 3 and 4 μg⋅kg^–1^, respectively (10). Adding midazolam or ketamine by oral or nasal administration route augments the sedative effect and increases the success rate (11–14). However, additional administration of these medications is limited by the oral-administration-induced low bioavailability and potential damage to nasal nerves and mucosa by their low *pH*.

A limited number of studies exist describing the combination of intranasal dexmedetomidine with oral/intranasal midazolam for MRI sedation (11, 12, 15). Anecdotal evidence suggests that buccal midazolam is well tolerated, has a considerably higher bioavailability and has better therapeutic efficacy than oral midazolam in children (16). Our previous studies showed that intranasal dexmedetomidine combined with buccal midazolam outperformed oral chloral hydrate or intranasal dexmedetomidine alone in computerized tomography and auditory brainstem response studies (9, 17). In this prospective interventional study, we sought to investigate the efficacy and safety of intranasal dexmedetomidine in combination with low-dose buccal midazolam in a relatively large cohort of children. This study hypothesized that the combination of buccal midazolam with intranasal dexmedetomidine could be an efficient and safe non-parenteral paradigm for children undergoing MRI examinations.

## Materials and methods

A prospective, interventional study was conducted from January to May 2018 at Guangzhou Women and Children’s Medical Center. The study protocol was approved by the Clinical Research Ethics Committees (2017071201). The trial was registered at chictr.org.cn (Principal investigator: B. L. Li, Date of registration: 2017/11/10, No. ChiCTR-OPC-17013328) prior to patient recruitment. After a detailed explanation by a pediatric anesthesiologist, each child’s statutory guardian provided written informed consent. Children between 1 month and 10 years old with an American Standards Association physical status of I–III requiring sedation for a short-duration MRI were enrolled. A short-duration MRI is defined as an MRI less than 1 h in duration.

The exclusion criteria included patients with a corrected postnatal age of less than 28 days, an active upper respiratory infection, airway malformations or a history of significant airway obstruction (i.e., heavy snoring, obstructive sleep apnoea, macroglossia, and micrognathia), and diagnoses of cleft lip and cleft palate. In addition, patients under current treatment with digoxin, alpha-adrenergic or beta-adrenergic agonists or antagonists, known allergies to dexmedetomidine and/or midazolam, history of paradoxical reactions for midazolam, existing bradycardia and/or hypotension, or patients having received any other sedative within the previous 48 h were also excluded.

To meet the requirement for pediatric MRIs, our medical center developed a pediatric sedation service program directed by anesthesiologists. Pediatric anesthesiologists performed pre-sedation assessments, failed sedation rescue, management of patients with an ASA greater than III, as well as general anesthesia. They recorded the time for sleep deprivation as well as a history of allergies, surgery, sedation, and sedation failure. Specially trained sedation nurses were responsible for preparing and administering the medications, monitoring vital signs in patients with ASA physical status II or below, following up with the patient post-sedation and recording data.

After the pre-sedation assessment, the buccal mucosa and nasal passages were dried and cleaned with a cotton swab before drug administration. A sedation nurse administered 0.2 mg⋅kg^–1^ midazolam hydrochloride injection at a concentration of 5 mg⋅mL^–1^ (Li Yue Xi^®^, Nhwa Pharma Corporation, Jiangsu, China) by buccal route, followed by 3 μg⋅kg^–1^ dexmedetomidine hydrochloride injection at a concentration of 100 μg⋅mL^–1^(Ai Bei Ning^®^, Jiang Su Heng Rui Medicine Co., Ltd., Lian Yun Gang, China) by nasal route to the recruited children. In our experience, buccal administration of medication is more acceptable than nasal administration. Accordingly, buccal midazolam is usually given first to increase patient cooperation. Given that buccal midazolam formulation is currently unavailable in our country, we prepared palatable buccal midazolam by intravenous midazolam hydrochloride formulation combined with an equivalent volume of medical-grade sucrose syrup (Guangdong Bang Min Pharmaceutical Co., Ltd., Lian Yun Gang, Guangzhou, China). The bioavailability of midazolam after buccal administration is mainly dependent on buccal exposure time (18). To increase the duration of buccal exposure, the use of sticky sucrose syrup improved the bitter taste and enhanced the adhesion of the water-soluble midazolam hydrochloride injection to the buccal mucosa. With a 1 ml needleless sterilized syringe, the mixed buccal solution was dripped and smeared evenly over the buccal mucosa, followed by 100 μg⋅ml^–1^ preservative-free and undiluted dexmedetomidine divided equally between nostrils *via* nasal atomizers (MAD Nasal TM, Teleflex Incorporated, United States). The participants were encouraged to remain in the supine position for 1–2 min to prevent spillage and ensure nasal drug absorption.

Oxygen saturation, pulse rates, sedation behaviors, and sedation scores were assessed every 5 min, whereas non-invasive blood pressure was documented by dedicated research coordinators every 10 min during the study. The MRI was obtained with a Siemens Skyra 3.0 Tesla scanner (Siemens, Erlangen, Germany) with an average acoustic noise of around 88 decibels. A single radiologist graded real-time movement artifacts with a four-point movement score after the image was obtained (Table 1).

**TABLE 1:** Evaluation scales.

Behavior scores	
**1**	Calm and cooperative
**2**	Anxious but reassurable
**3**	Anxious and not reassurable
**4**	Crying, or resisting
**University of Michigan Sedation Scale (UMSS)**	

**0**	Awake/Alert
**1**	Minimally sedated: tired/sleepy, appropriately responds to verbal conversation and/or sounds
**2**	Moderately sedated: somnolent/sleeping, easily aroused with light tactile stimulation
**3**	Deeply sedated: deep sleep, arousable only with significant physical stimulation
**4**	Unarousable
**Movement scores**	

**1**	Not moving
**2**	Involuntary mild body moving
**3**	Involuntary moderate body moving
**4**	Purposeful body moving

If a child did not achieve a University of Michigan Sedation Score (UMSS) of 2 or above within 30 min after the initial sedation regimen, or the patient woke up (UMSS 0–1) and could not complete the MRI, a rescue dose of 2 μg⋅kg^–1^ intranasal dexmedetomidine was administered. These children were identified as having an initial sedation failure. Rescue sedation was managed and monitored by a pediatric anesthesiologist. If rescue medication failed, patients were transitioned to general anesthesia or rescheduled.

**FIGURE 1:**
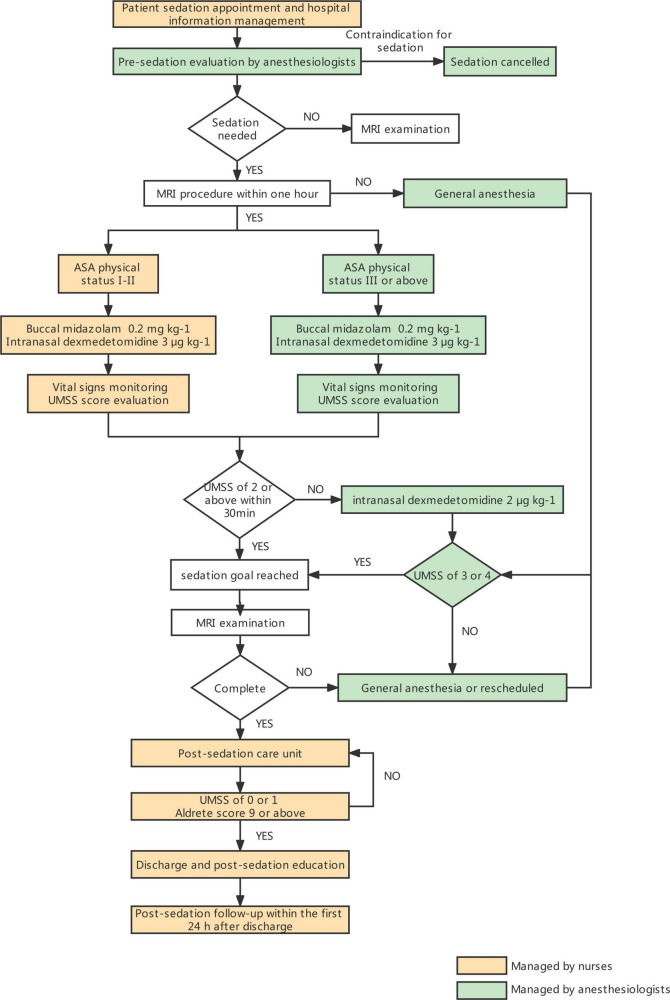
Flow diagram of sedation for magnetic resonance imaging.

Tactile stimulation was employed after the MRI to induce post-sedation wake-up. If the UMSS remained in the range of deep sedation (UMSS of 3 to 4) 15 min after the assessment, washcloths were used to wipe the face. Patients were required to have a UMSS of 0 or 1, an Aldrete score of more than 9 and the capacity to consume a fluid drink prior to discharge. A post-sedation survey was explained and delivered to the parents or guardians to quantify the level of consciousness and time to resume normal activities (the time to resume eating, walking and playing as usual) within the first 24 h after discharge. Sedation-related side effects, such as restlessness, hyperactivity and agitation, motor imbalance/fall, respiratory difficulties, gastrointestinal upset and the need for medical consultation were also collected. A nurse contacted the parents or guardians by phone and completed the survey on the day following sedation. Figure 1 shows the flow diagram of sedation for MRI.

The primary outcome was successful sedation, which was defined as children with UMSS of 3–4 within 30 min after the initial drug administration and completion of the MRI examination. Meanwhile, the risk factors for failed sedation, behavior scores at the time of intranasal and buccal drug delivery, onset time, movement scores during the procedure, times to wake up and discharge, adverse events, post-sedation side effects, and the time from discharge to resume normal activities were all included as secondary outcomes. Acceptable behaviors were defined as behavior scores of 1–2, whereas poor behaviors were defined as a behavior score of 4. The time required to attain a UMSS of 2 following the initial dose of the drug was considered the onset of sedation. Waiting time was defined as the time between the beginning of sedation and the start of MRI, whereas wake-up time was defined as the time between the drug administration and the child’s return to a UMSS of 0 or 1 following sedation. Prolonged sedation was documented if the patient could not achieve discharge criteria one hour after the completion of the MRI examination. Patients who had pulse rate and non-invasive blood pressure 20% lower or higher than the age-defined normal range limits were recorded (19). Hypoxia was defined as SpO_2_ of 93% or below in the patients without supplemental oxygen.

## Statistical analysis

On the basis of a 90% success rate on the auditory brainstem response test by intranasal dexmedetomidine with buccal midazolam (9), the success rate for MRI was defined as at least 4% higher than that value, and 498 children were required to be detected with a 90% power and a two-sided significance level of 0.05. In consideration of a lost-to-follow-up rate of about 6%, 530 patients were needed in this study. Numeric variables such as demographic characteristics, adverse events, and categorical data were presented as median (interquartile range) or frequencies (percentage). The Chi-square test, t-test or Mann–Whitney U test was employed to evaluate the demographic characteristics of the successful and failed sedation groups. Multivariate logistic regression was performed to identify each value as a contributing factor to failed sedation. Correlation analysis was conducted on age and onset time, age and wake up time, age and discharge time, duration of the procedure and wake up time, and duration of the procedure and discharge time. SPSS 20.0 (SPSS Inc., Chicago, IL, United States) was used for statistical analyses, and the R package (Vienna, Austria) was utilized to plot figures. A two-sided *P* < 0.05 was considered statistically significant.

## Results

From January through May of 2018, 2750 children were recruited, with 533 of them being enrolled and sedated. The guardians of 1,067 patients refused to participate in the trial, whereas 547 patients preferred different sedative techniques. Three children withdrew following drug administration: one patient after neurosurgery developed a high fever, causing the MRI to be canceled and rescheduled; the other two withdrew due to parental refusal of data collection. Eventually, 530 children were administered dexmedetomidine–midazolam sedation and included in the final analysis. Table 2 summarizes the demographic information and clinical indications for MRI examinations.

**TABLE 2:** Subjects’ characteristics and procedures. Values in number (%) or median (IQR [range]).

	Overall	Successful sedation	Failed sedation	*P*- value
	**(*n* = 530)**	**(*n* = 505)**	**(*n* = 25)**	
Gender, Male	358 (67.5%)	338 (66.9%)	20 (80.0%)	0.173
Age (mo)	23 (7–43 [1–132])	22 (7–43 [1–132])	25 (11–46 [1–108])	0.312
Body Weight (kg)	11 (7.5–15 [3-40])	11 (7.5–15 [3–40])	12 (9–14 [4–33])	0.323
ASA				0.323
I	66 (12.5%)	61 (12.1%)	5 (20.0%)	
II	386 (72.8%)	371 (73.5%)	15 (60.0%)	
III	78 (14.7%)	73 (14.5%)	5 (20.0%)	
History of sedation failure	105 (19.8%)	71 (14.1%)	10 (24.4%)	0.001
History of surgery	74 (14.0%)	69 (13.7%)	5 (20%)	0.551
History of sedation	307 (57.9%)	289 (57.4%)	18 (72%)	0.144
Time awake before sedatives administration (h)	4 (2.5–5.5 [0–12])	4 (2.5–5.5 [0–12])	4 (2–5 [0.5–8])	0.455
**MRI examination sites classification**				
Brain	442 (83.4%)	425 (84.2%)	17 (68%)	
Chest	7 (1.3%)	7 (1.4%)	0 (0%)	
Abdomen	24 (4.5%)	22 (4.4%)	2 (8%)	
Lumbar	9 (1.7%)	8 (1.6%)	1 (4%)	
Biliary and Urinary tract system	27 (5.1%)	24 (4.8%)	3 (12%)	
Limbs	15 (2.8%)	14 (2.8%)	1 (4%)	
Spinal cord	1 (0.2%)	1 (0.2%)	0 (0%)	
Multiple sites	5 (0.9%)	4 (0.8%)	1 (4%)	
**Diagnostic categories**				
Neurosurgery	120 (22.6%)	116 (23%)	4 (16%)	
Neurobehavioral disease	132 (24.9%)	127 (25.1%)	5 (20%)	
Motor/language retardation	77 (14.5%)	72 (14.3%)	5 (20%)	
Epilepsy	59 (11.1%)	57 (11.3%)	2 (8%)	
Hepatitis syndrome	34 (6.4%)	33 (6.5%)	1 (4%)	
Urology	21 (4.0%)	17 (3.4%)	4 (16%)	
Endocrinology	19 (3.6%)	19 (37.6%)	0 (0%)	
Orthopedics	19 (3.6%)	17 (3.7%)	2 (8%)	
Ophthalmology	9 (1.7%)	9 (1.8%)	0 (0%)	
Vascular disease	8 (1.5%)	8 (1.6%)	0 (0%)	
Others	32 (6.0%)	30 (5.9%)	2 (8%)	

Furthermore, 505 out of 530 [95.3% (95% confidence interval (CI): 93.5–97.1%)] children underwent satisfactory sedation and completed MRI examination with the initial sedation regimen. Meanwhile, 13 of the 25 initial sedation failures were successfully sedated after the treatment with rescue intranasal dexmedetomidine. This non-parenteral sedation regimen allowed 518 children [97.7% (95% CI 96.5–99%)] to complete the MRI examination, with 8 children transitioning to propofol anesthesia and four children being rescheduled. During the MRI examinations, 503 of 505 (99.6%) initially successfully sedated patients had movement scores of 2 or below. Buccal midazolam and intranasal dexmedetomidine were successfully administered to all patients. A total of 261 (49.2%) and 319 (60.2%) of the 530 children had acceptable behavior scores with intranasal and buccal administration, respectively (Table 3).

**TABLE 3:** Secondary outcomes.

Outcomes	Values(n = 530)
Movement scores*	
1	387 (76.6%)
2	105 (20.8%)
3	12 (2.4%)
4	1 (0.2%)
**Behavior with nasal drug administration^†^**	
1	154 (29.1%)
2	107 (20.2%)
3	127 (24.0%)
4	147 (27.7%)
**Behavior with buccal drug administration^†^**	
1	212 (40%)
2	107 (20.2%)
3	93 (17.5%)
4	118 (22.3%)
Onset time (min)	10 (10–15 [3–30])
Waiting time (min)	12 (5–20 [0–75])
Wake-up time (min)	45 (35–55 [12–142])
Discharge time (min)	50 (40–60 [15–147])
Duration of MRI (min)	15 (10–20 [10–60])
Time to resume normal activities (h)	5.5 (4–8 [0.8–20.6])

Values in number (%) or median (IQR [range]).

*Movement scores: 1. Not moving, 2. Involuntary mild body moving, 3. Involuntary moderate body moving, 4. Purposeful body moving.

^†^Behavior scores: 1. Calm and cooperative, 2. Anxious but reassurable, 3. Anxious and not reassurable, 4. Crying, or resisting.

**FIGURE 2:**
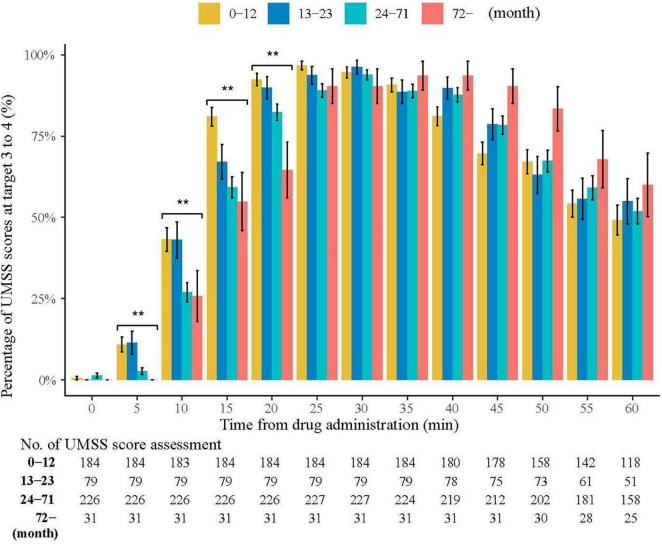
Percentage of UMSS Scores at Target 3 to 4. University of Michigan Sedation Scores (UMSS) range from 3 to 4 with four different age categories. In the first 60 min after drug administration, 95.5% of children with UMSS reached the target of deep sedation (3 to 4). The percentages of UMSS in deep sedation for 5, 10, 15, and 20 min were significantly different among the four age groups (*P* = 0.001, *P* = 0.002, *P* < 0.001, and *P* < 0.001, respectively). There was a significant increasing trend in the onset time with age in months (*R* = 0.249, *P* < 0.001). The wake-up time and discharge time were significantly correlated with duration of procedure (*R* = 0.323, *P* < 0.001 vs. *R* = 0.325, *P* < 0.001). **P < 0.01.

Table 3 shows the sedation and procedure times. Three patients experienced prolonged sedation (65, 75 and 125 min) during recovery. The age categories in this study were consistent with those recommended by the American Academy of Pediatrics for Clinical Trials (20). On a monthly basis, a significant rising trend was observed in the onset time concerning age (*R* = 0.249, *P* < 0.001). In the first 60 min after drug administration, 95.5% of children with UMSS reached the target of deep sedation (3 to 4). The percentages of UMSS at 3 to 4 for 5, 10, 15, and 20 min were significantly different among the four age groups (*P* = 0.001, *P* = 0.002, *P* < 0.001, and *P* < 0.001, respectively). The wake-up and discharge times were significantly correlated with the duration of the procedure (*R* = 0.323, *P* < 0.001 vs. *R* = 0.325, *P* < 0.001). No statistical correlation was observed between age and wake-up time (*R* = −0.054, *P* = 0.223), nor between age and discharge time (*R* = −0.833, *P* = 0.061) (Figure 2).

In terms of the different parameters evaluated, only the history of failed sedation was considered a statistically significant risk factor for failed sedation with the current regimen in the univariate logistic regression model [odds ratio = 2.88 (95% CI: 1.25–6.60), *P* = 0.013] and multivariate logistic regression models [odds ratio = 4.71 (95% CI: 1.24–17.87), *P* = 0.023] (Table 4).

**TABLE 4:** Factors contributing to sedation failure.

Variables	Univariable				Multivariable		

	Odds ratio	95%CI	P-value	Odds ratio		95%CI	*P-*value
History of sedation failure	2.88	1.25–6.60	0.013	4.71		1.24–17.87	0.023
Poor behaviors with buccal drug administration	0.87	0.32–2.36	0.781	0.32		0.03–3.83	0.371
Poor behaviors with nasal drug administration	0.86	0.34–2.19	0.747	1.57		0.19–13.05	0.678
Neurobehavioral disease	1.01	0.42–2.48	0.976	1.91		0.52–6.96	0.329
History of surgery	1.58	0.57–4.35	0.376	2.11		0.58–7.67	0.259
History of sedation	1.92	0.79–4.68	0.151	0.66		0.16–2.64	0.554
Awake time before sedation less than 4 hours	1.09	0.48–2.46	0.845	1.16		0.36–3.75	0.807
Duration of MRI examination	1.04	0.99–1.08	0.098	1.03		0.98–1.08	0.212
**Age, months**							
1–12	Ref			Ref			
13–23	0.23	0.03–1.80	0.161	0.00		0.00–2.34	0.952
24–71	1.05	0.45–2.45	0.909	0.72		0.21–2.51	0.607
71–120	0.57	0.07–4.62	0.599	0.57		0.07–4.98	0.973

During the onset of sedation, two patients vomited, and one became agitated. Hypotension and bradycardia occurred in 35 (6.6%) and 12 (2.3%) patients, respectively. Four patients (0.75%) had a temporary blood pressure decrease below 30% of the normal range of the age groups, and seven patients experienced tachycardia. All adverse reactions were transient self-limiting and returned to normal after the patients awakened. No children required oxygen supplementation or medication intervention nor had concurrent hypotension and bradycardia during the study.

**FIGURE 3:**
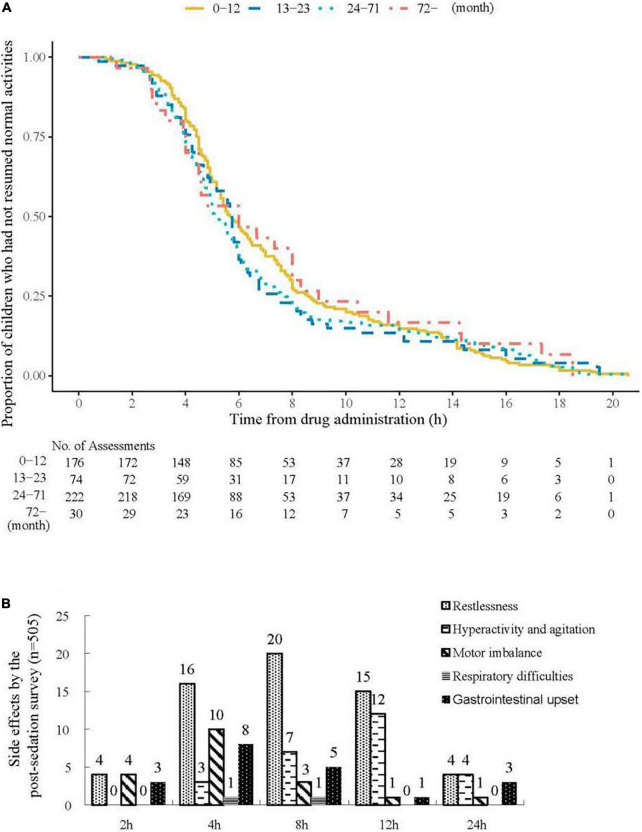
Time to resume normal activities and side effects by the post-sedation survey. **(A)** Time to resume normal activities after drug administration in different age groups. The median (interquartile range) time to resume normal activities was 5.5 (4–8) h. No statistical differences were observed among different age groups for the time to resume normal activities (*P* = 0.724). **(B)** Side effects by the parental feedback in the follow-up survey.

A total of 500 patients (94.3%) responded to the post-sedation survey the next day after sedation. The median time to resume normal activities was 5.5 h. No statistical differences were observed among different age groups regarding the time to resume normal activities (*P* = 0.724, Figure 3A). In the follow-up survey, parents reported 56 (11.2%), 25 (5.0%), 19 (3.8%), and 17 (3.4%) instances of restlessness, hyperactivity and agitation, unsteadiness, and gastrointestinal upset, respectively. No children reported having respiratory problems nor requiring extra medical assistance (Figure 3B).

## Discussion

In this prospective interventional study, we revealed that buccal midazolam combined with intranasal dexmedetomidine was associated with a high success rate in short-duration MRI examinations. This non-parenteral sedation protocol was ensured adequate sedation in 97.7% of children between 1 month and 10 years old with a smooth hemodynamic status and no serious adverse events.

We have been investigating a convenient and effective sedation regimen to manage anxiety and minimize psychological trauma in children having MRI examinations. Recent advancements in the administration of dexmedetomidine–midazolam combination therapy revealed that these two drugs combination provided an alternative for MRI sedation in children (11–13, 21). Success rates of 82.5–84% were obtained using intranasal dexmedetomidine combined with oral midazolam (11, 13), whereas success rate of 88–95% was observed when utilizing intranasal dexmedetomidine–midazolam combination (12, 22). Nevertheless, low *pH* and undiluted midazolam is associated with unpleasant burning sensation and may eaisly disrupt nasal mucosa and nerves. Hence, intranasal midazolam is not recommended in pediatric sedation. The bioavailability of the oral midazolam is 21%, and that of buccal midazolam ranged from 43.6% to 66.1% (18). We employed buccal administration instead of intranasal or oral midazolam to avoid irritability with good bioavailability. Although the bioavailability of buccal midazolam is mainly dependent on buccal exposure time (18), the success rate of 97.7% was higher than that of the combination with intranasal or oral midazolam, which the efficacy was comparable to that of intravenous propofol (23, 24).

Dexmedetomidine is distinguished by its capability to induce deep sedation that closely resembles physiological sleep. Nevertheless, patients can be easily and unpredictably aroused by sounds or movements, which always cause serious interruptions in the MRI and prolong examinations in a busy setting (23). The addition of intranasal/oral ketamine can enhance intranasal dexmedetomidine-induced sedation efficacy and shorten sedation onset time (25, 26). However, this regimen is associated with a large volume of intranasal administration. The possible ceiling effect of large volume intranasal administration may lead to swallowing of administered drug and reduced bioavailability. Our data suggested that the combination of buccal midazolam to intranasal dexmedetomidine increased the depth of sedation while still allowing full awakening with stimulation during the recovery period. A total of 99.6% subjects had acceptable behaviors during the MRI examinations. This pharmacological property indicated that the dexmedetomidine–midazolam combination could prevent unexpected patient movements from interfering with the MRI procedure and yet they could still be easily roused after examinations. This unique characteristic was revealed by a positive correlation between wake-up and discharge time with duration of the procedure. Compared with the 30% success rate by utilizing intranasal dexmedetomidine alone at the same dose, this protocol produced an initial success rate of 95.3% (10). The success rates of other traditional non-parenteral sedatives in MRI sedation were 86.7% for chloral hydrate in children aged 0 to 10 years (27), 67% for pentobarbital in children aged 8 months to 8 years (28), and 59% for midazolam in children aged 1 to 7 years (29). A previous study revealed that the success rate of dexmedetomidine sedation varied with age, weight and disease (30). In the current study, we have shown that history of sedation failure was the only risk factor contributing to sedation failure and this finding was consistent with Liu’s study on intranasal dexmedetomidine–ketamine combination (31).

Safety is the priority in pediatric MRI sedation. Dexmedetomidine and midazolam work on different receptors that results in a synergistic sedation effect (32). Moreover, since a lower dose of dexmedetomidine is required to achieve the same level of sedation, fewer hemodynamic adverse effects are observed (33). Compared with monotherapy using dexmedetomidine, the multimodal approach of a two-sedative treatment produced greater respiratory control and hemodynamic stability (31). Another retrospective study has also shown that the addition of low-dose dexmedetomidine to propofol-based sedation was associated with decreased need for airway support and improved hemodynamic stability (34). The success rate of intravenous dexmedetomidine–midazolam anesthesia is comparable to that of propofol anesthesia, however, bradycardia occurs more frequently than in the case of propofol anesthesia (35, 36). Intriguingly, the intranasal and buccal routes demonstrated delayed serum concentrations with lower peak plasma concentration than that achieved by intravenous administration, hence this may reduce the risk of adverse events (37). When intranasal dexmedetomidine and oral midazolam were used in MRI examinations, hypotension and bradycardia occurred in 3 and 8% of children, respectively (11). Although the incidences of bradycardia (2.3%) and hypotension (6.6%) in our study were higher than that of the intranasal dexmedetomidine–ketamine combination (0.02%) (25), all the hemodynamic adverse reactions were transient and self-limiting, and no major adverse events nor emergent airway intervention occurred in the current study. However, the frequency of delirium and related behaviors alteration after MRI sedation with ketamine or midazolam as an adjunctive agent warrants further clarification.

Although intranasal dexmedetomidine is mild and non-irritating, buccal administration was still more acceptable than nasal administration as illustrated by an increase in calm and cooperative behaviors during administration. Patients with neurological dysfunction undergoing regular brain scans accounted for half of the patients included in this study. It was consistent with our previous study of children with autism that patients with neurological dysfunction should be the main factor affecting behavior scores at drug administration (17). However, successful administration of the buccal and nasal drug was delivered to all patients without secondary supplement. Nausea and vomiting are common side effects associated with the use of non-parenteral sedatives. In our study, 0.4% of patients vomited during the procedure, and 3.4% reported gastrointestinal upset in the 24-h post-sedation survey. This value was lower than that of adverse events observed with oral chloral hydrate and pentobarbital (30). A wide distribution was observed during the time to resume normal activities in the survey. The reason for the prolonged recovery time could be secondary to reporting bias from parents. Regarding the 0.4–0.8% risk of safety adverse events during the sedation of pediatric patients for imaging examinations (5, 38), a larger sample size may be required to identify the incidence of serious adverse events.

The median sedation onset time (10 min) of this study was shorter than that in previous dexmedetomidine–midazolam research (15 min) (9, 17). The reason should be the utilization of a double dosage of midazolam based on a deeper level of sedation required by MRI examination. The higher dose of midazolam may have accelerated the onset time. A rising trend was observed in the onset time concerning age in our study. This onset time was similar to that of Sun’s study (9.6 min) in children between 1 month and 36 months (39) but shorter than those of other studies on intranasal dexmedetomidine combinations with intranasal/oral ketamine (15–17 min) in children between 0 months and 7 years old (14, 25, 26, 31). Compared with propofol, this dexmedetomidine-midazolam regimen is time-consuming and requires appropriate time allotment in a high-patient-volume setting. However, it reduces the workload of pediatric anesthesiologists and the cost of medical care in a vast pediatric population.

Certain limitations in this study should be considered. First, this study is a one-arm prospective trial with no randomization. We previously showed that dexmedetomidine–midazolam combination performed better in auditory brainstem response testing and computed tomography than chloral hydrate or intranasal dexmedetomidine alone (9, 17). This study was designed to focus on the efficacy and safety of this non-parenteral sedation paradigm in a relatively large population of children undergoing MRI examinations. In addition, given that the regular brain scan is the principal procedure in our radiology department, the mean MRI examination time is relatively short in this cohort. To enhance recovery, we usually woke patients up after the examinations rather than waiting for a natural awakening. Hence, the wake-up and discharge times were correlated with the duration of the procedure and assumed a relatively quick wake-up period. However, the duration of MRI examination was not a significant risk factor for failed sedation with the current regimen. Furthermore, although the post-sedation surveys were comprehensively explained before patient discharge, bias by guardians were still unavoidable and might have caused a variation in the survey information. Lastly, since buccal midazolam formulation is currently unavailable in our country, we employed a combination of midazolam injection formula with sucrose syrup to promote drug adherence. However, some portion of drug must be swallowed and resulting in reduced bioavailability.

## Conclusion

This study indicated that the addition of low-dose buccal midazolam to intranasal dexmedetomidine administration was associated with a high success rate in children between 1 month and 10 years undergoing short-duration MRI examinations. Compared with intravenous access, the advantages of this non-parenteral regimen include easy operation with a good safety profile. Future studies should focus on the development of buccal and intranasal drug formula to shorten the onset time and characterization of the pharmacokinetics profiles of these non-parenteral administration.

## Data availability statement

The datasets presented in this study are available in online repositories (https://pan.baidu.com/s/1EbV62svLFaFHuAOe3dAwSA?pwd=4338) or from the corresponding author upon reasonable request.

## Ethics statement

The studies involving human participants were reviewed and approved by Guangzhou Women and Children’s Medical Center. Written informed consent to participate in this study was provided by the participants’ legal guardian/next of kin.

## Author contributions

BL and VY designed the study, analyzed data, wrote the most of draft of the manuscript, and performed revision. BL, HL, HZ, and JH performed patient recruitment, data gathering, and data analysis. BL, VY, JP, and XS revised the manuscript. All authors read and approved the final manuscript.
